# A Systematic Design Optimization Approach for Multiphysics MEMS Devices Based on Combined Computer Experiments and Gaussian Process Modelling

**DOI:** 10.3390/s21217242

**Published:** 2021-10-30

**Authors:** Shayaan Saghir, Muhammad Mubasher Saleem, Amir Hamza, Kashif Riaz, Sohail Iqbal, Rana Iqtidar Shakoor

**Affiliations:** 1 Department of Mechatronics Engineering, National University of Sciences and Technology (NUST), Islamabad 44000, Pakistan; shayaan.saghir18@mts.ceme.edu.pk (S.S.); a.hamza@ceme.nust.edu.pk (A.H.); 2 National Centre of Robotics and Automation, Islamabad 44000, Pakistan; rana.iqtidar@mail.au.edu.pk; 3 Department of Electrical Engineering, Information Technology University of the Punjab (ITU), Lahore 54600, Pakistan; kashif.riaz@itu.edu.pk; 4 Department of Mechanical and Aerospace Engineering, Air University (AU), Islamabad 44000, Pakistan; sohail.iqbal@mail.au.edu.pk; 5 Department of Mechatronics Engineering, Air University (AU), Islamabad 44000, Pakistan

**Keywords:** microelectromechanical systems (MEMS), design and analysis of computer experiments (DACE), Gaussian process (GP), finite element method (FEM), multiphysics, optimization

## Abstract

This paper presents a systematic and efficient design approach for the two degree-of-freedom (2-DoF) capacitive microelectromechanical systems (MEMS) accelerometer by using combined design and analysis of computer experiments (DACE) and Gaussian process (GP) modelling. Multiple output responses of the MEMS accelerometer including natural frequency, proof mass displacement, pull-in voltage, capacitance change, and Brownian noise equivalent acceleration (BNEA) are optimized simultaneously with respect to the geometric design parameters, environmental conditions, and microfabrication process constraints. The sampling design space is created using DACE based Latin hypercube sampling (LHS) technique and corresponding output responses are obtained using multiphysics coupled field electro–thermal–structural interaction based finite element method (FEM) simulations. The metamodels for the individual output responses are obtained using statistical GP analysis. The developed metamodels not only allowed to analyze the effect of individual design parameters on an output response, but to also study the interaction of the design parameters. An objective function, considering the performance requirements of the MEMS accelerometer, is defined and simultaneous multi-objective optimization of the output responses, with respect to the design parameters, is carried out by using a combined gradient descent algorithm and desirability function approach. The accuracy of the optimization prediction is validated using FEM simulations. The behavioral model of the final optimized MEMS accelerometer design is integrated with the readout electronics in the simulation environment and voltage sensitivity is obtained. The results show that the combined DACE and GP based design methodology can be an efficient technique for the design space exploration and optimization of multiphysics MEMS devices at the design phase of their development cycle.

## 1. Introduction

MEMS accelerometers are miniaturized sensors for measuring constant, time varying, and quasi-static accelerations and have wide applications in the field of automotive industry [1], machine condition monitoring [2,3], shock sensing [4], precision navigation [5], and consumer electronics [6]. For these applications, one basic requirement is that the MEMS accelerometer must be able to determine the position of a body in space by sensing its acceleration in three axes. This is generally achieved by designing a single axis MEMS accelerometer and mounting orthogonal to each other on a body for acceleration measurement. This results not only increased device footprint area and increased packaging cost, but also measurement error due to misalignment [7]. To resolve these issues, one of the solutions reported in the literature is the monolithic integration of three proof masses, each sensing in a specific axis, in a single chip [8,9,10]. Though, this approach minimizes the packaging cost, but achieving small device size, high sensitivity and small noise floor values remain the main limitations. In comparison to multiple proof masses integrated, MEMS accelerometers based on single proof mass for sensing multiple axis acceleration have proved to be an efficient solution for achieving a small device footprint, low cost, and improved performance [11,12,13].

MEMS are multiphysics devices and generally require the optimization of multiple output responses for a given set of design parameters and microfabrication process constraints. The optimization of MEMS is traditionally carried out by varying one factor at a time and analyzing its effect on an output response either by developing analytical models, FEM models, or topology optimization [14,15,16,17,18,19]. These methods are efficient for MEMS devices with simple geometric configuration but for MEMS with relatively complex geometry and with the requirement of multiphysics design space exploration and multiple output responses to be optimized simultaneously, these methods become inefficient due to high computational costs and modelling complexity.

The optimization of a capacitive MEMS accelerometer design is a multiphysics problem involving electro–thermal–structural interactions. The main performance responses of a capacitive MEMS accelerometer that must be optimized include natural frequency, proof mass displacement, pull-in voltage, capacitance change, and thermomechanical noise. The natural frequency and proof mass displacement are strongly dependent on the stiffness of mechanical suspension beams. For high sensitivity, the natural frequency value should be low which requires low stiffness mechanical suspension beams or large proof mass. However, low mechanical stiffness of suspension beams leads to a low value of pull-in voltage which further limits the maximum value of initial bias voltage that can be applied to the sensing capacitive parallel plates and maximum input acceleration range. Similarly, for high resolution, a large value of capacitance change is desired in a MEMS accelerometer which is dependent on the initial air gap between the sensing parallel plates and their overlap area. A large value of overlap area and small air gap results in large capacitance change but decreases the pull-in voltage threshold. Moreover, the air damping between the sensing parallel plates increases by increasing the overlap area of plates and decreasing their initial gap. Thus, decreasing the quality factor and hence increasing the thermomechanical noise. Thus, it becomes important that for the geometric design optimization of MEMS accelerometer, all the output responses be considered simultaneously instead of the traditional approach of varying one factor at a time and analyzing its effect on a single output response presented in the literature [20,21,22].

The design of experiments (DoE) is a systematic statistical approach for optimization problems and has been a widely used optimization technique in many different fields, for example in manufacturing processes [23], sensor optimization [21,24], and precision agriculture [25]. The traditional DoE approach explores the design space of an optimization problem with a minimum number of physical experiments and the metamodels for output responses are developed by estimating the effect of design parameters using least square regression which is based on the randomness due to experimental variations [26]. Previously, the authors have presented the application of traditional DoE based optimization methodology for MEMS devices [27,28]. The application of the traditional DoE technique based on least square regression for deterministic computer simulations has been a matter of discussion in the literature since it lacks random error and the method of least squares residuals has no statistical meaning [29]. However, Simpson et al. [30] have argued that the application of traditional DoE for computer simulations is a trade-off of appropriateness vs. practicality. Sacks et al. [31] pointed out that the selection of design points for deterministic computer simulations is a statistical experimental design problem and also proposed a Gaussian process (GP) model by considering the deterministic output responses as random stochastic processes.

The design and analysis of computer experiments (DACE) based on GP models are now a popular choice for the development of metamodels, using deterministic computer simulations, in different fields and allow the design space exploration with minimum computational cost [32,33,34,35]. For MEMS devices, the DACE based design optimization, combined with the FEM based MEMS simulation tools and latest high computing machines, can greatly minimize the MEMS development cycle time and reduce the costs involved in the traditional iterative microfabrication runs for the realization of a fully functional MEMS device.

In this paper, we present a DACE based systematic and efficient design methodology for MEMS in general and MEMS accelerometer in particular by using Latin hypercube sampling (LHS) technique to create a design space with different combinations of geometric design parameters and GP based metamodelling for the multi-response optimization. The optimization for the MEMS accelerometer is carried out by considering the microfabrication process constraints and environmental operating conditions.

## 2. Working Principle and Structural Design of MEMS Accelerometer

**Figure 1:**
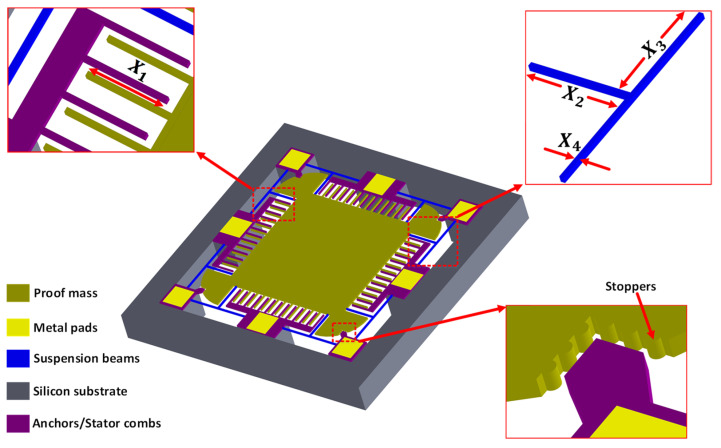
Schematic of the single proof mass 2-DoF MEMS capacitive MEMS accelerometer.

Figure 1 shows the schematic of the proposed two degree-of-freedom (2-DoF) MEMS accelerometer design conceptualized considering the constraints imposed by the commercially available SOIMUMPs microfabrication process offered by MEMSCAP Inc. USA [36]. The main constraints of the SOIMUMPs process are (a) no bottom electrode is available for the out-of-plane proof mass displacement sensing and (b) the design cannot have anchors in the middle of the proof mass, thus limiting the position of the mechanical springs to the outer side of the mass. For sensing the proof mass displacement, corresponding to an input acceleration, parallel plate combs are attached to each of the four sides of the proof mass. The stator combs are located between the two rotor combs, attached to the proof mass, in a gap–antigap configuration. To obtain maximum capacitance change, the ratio of the larger gap to the smaller gap between combs is kept at 3 with gap and antigap values of 2.5 µm and 7.5 µm, respectively. The suspension beams are T-shaped and are designed symmetrically on the four corners of the proof mass to minimize the cross-axis coupling and increase stability. To minimize the effect of sudden high acceleration values, end stoppers are designed on the proof mass corners. The capacitance sensing mechanism is based on the gap closing mechanism and both stator and rotor combs are attached in differential sensing configuration to obtain the maximum capacitance change. The thickness of the structural silicon-on-insulator (SOI) layer in the whole structure is 25 μm.

## 3. Design and Analysis of Computer Experiments (DACE) Based Multi-Response Optimization

### 3.1. Gaussian Process Modelling

A generalized form of Gaussian distribution is termed as Gaussian process (GP), which is a type of continuous stochastic process. It is used to describe the probability distribution over functions and the model developed by GP is a conditional probabilistic model. When fitting data from a deterministic computer experiment, the Gaussian process model is frequently utilized. Sacks et al. [31] presented these models as models for computer experiments. They are desired because they give an exact fit to the data gathered from computer experiments and only need a single parameter for every design parameter. The optimization design space can be represented as $d_{s}=\left(x_{i},y_{i}\right)$ of k runs, where i=1, 2,…,k; $x_{i}$ is the D-dimensional vector of input design parameters for each run and $y_{i}$ is the scalar value of the output response. The design parameters and the output responses for the k runs can be written in a matrix $X={\left[x_{1},\;x_{2},\ldots \ldots ,\;x_{k}\right]}^{T}$ and $y={\left[y_{1},{\;y}_{2},\ldots \ldots ,{\;y}_{n}\right]}^{T}$, respectively. The Gaussian process model for an output response is defined as [26]:

(1) $$y_{i}=z\left(x_{i}\right)+\mu$$

where μ is the mean of the modelled surface and $z\left(x_{i}\right)$ is the Gaussian process, as a function of design parameters $x_{i}$. The value of output response from any collection of design parameters has a multivariate normal distribution, which defines a Gaussian process. This multivariate normal distribution has a mean $\mu 1_{n}$, where $1_{n}$ being the vector of ones of k length. The multivariate normal distribution also contains a covariance matrix that is proportional to a correlation matrix with a specific structure that allows the points to form a smooth surface. The covariance is:

(2) $$\mathrm{cov}\left(y\right)=\sigma^{2}R\left(X,\theta \right)$$

where $\sigma^{2}$ is the variance which is the proportionality constant between the covariance matrix and the correlation matrix. The matrix R(X,θ) represents the correlation matrix and is a function of hyperparameters θ and design parameters. The elements of the correlation matrix can be estimated using the relation [37]:

(3) $$r_{\mathrm{ij}}=\exp\left(-\overset{D}{\underset{t=1}{\sum }}\theta_{t}{\left(x_{\mathrm{it}}-x_{\mathrm{jt}}\right)}^{2}\right)$$

In Equation (3), $\theta_{t}\geq 0\;\left(t=1,\;2,\;\ldots ,\;D\right)$ the term $r_{\mathrm{ij}}$ specifies the correlation between the output responses at any two points. The correlation matrix is built so that the distance between any two different locations in the input domain is inversely proportional to the correlation of their outputs. In specifically, as the distance between the two input locations approaches zero, the correlation increases to one, and as the distance approaches infinity, the correlation decreases to zero.

**Figure 2:**
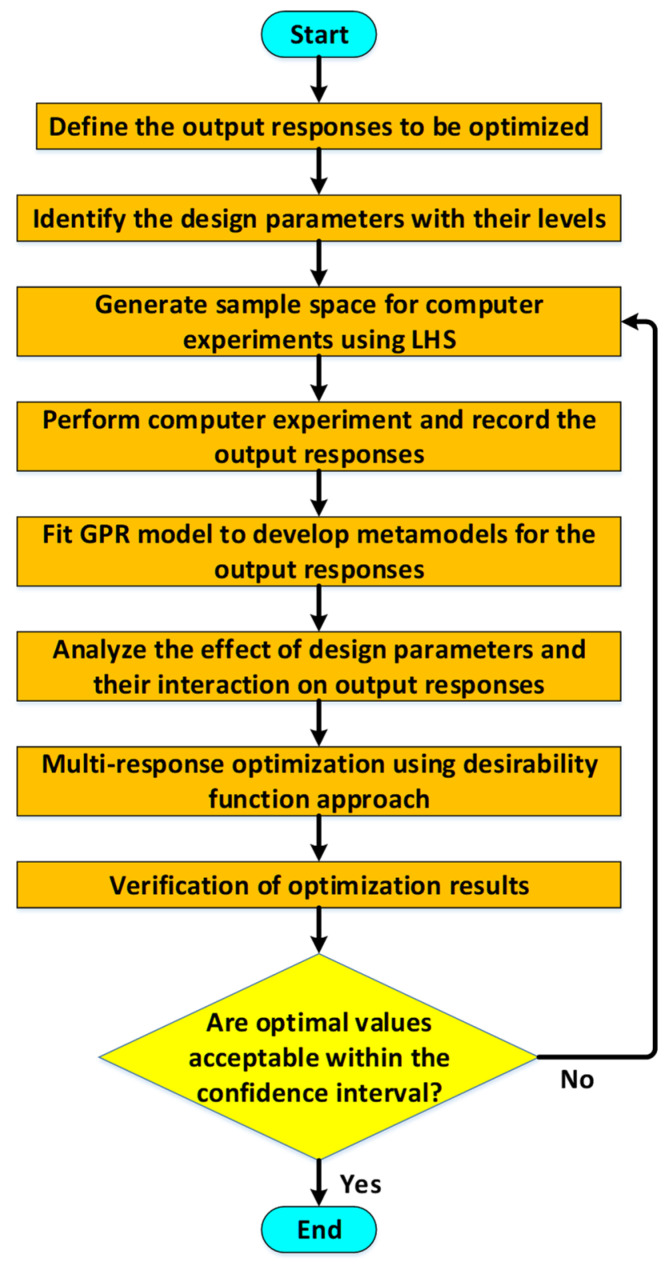
Flowchart for the DACE based optimization methodology.

The prediction equation to evaluate the value of an output response at other sets of design parameters is given as follows [38]:

(4) $$\hat{y}\left(x\right)=\hat{\mu }+r^{T}\left(x,\hat{\theta }\right)R^{-1}\left(X,\hat{\theta }\right)\left(y-\hat{\mu }1_{n}\right)$$

where $r^{T}=\left[r\left(x_{1},x\right),\;r\left(x_{2},x\right),\ldots ,r\left(x_{n},x\right),\right]$ and the values for $\hat{\theta }$ and $\hat{\mu }$ are used in Equation (4) after estimating the maximum likelihood estimates of parameters μ and θ. Figure 2 shows the DACE based optimization methodology implemented in this paper for the multi-response optimization of MEMS accelerometer. The first step is to identify the main output responses of the MEMS accelerometer to be optimized, which is followed by the selection of the geometric design parameters and operating conditions with the specification of low and high values for the design parameters. Since in the present study, the number of design parameters is eight, thus a statistical Latin Hypercube Sampling (LHS) based technique for the efficient sampling of the design points for the design parameters is implemented. The output responses for each simulation run in the LHS-based design matrix are obtained through Multiphysics FEM simulations. Based on the FEM simulation results for all the design points in the LHS based design matrix, a Gaussian process regression (GPR) analysis is performed and metamodels for all the output responses are obtained. The analysis of these metamodels for the output responses gives a detailed insight into the effect of the individual design parameters and their interaction on the output responses. The GPR analysis allows to analyze a single output response considering the design parameters but to simultaneously optimize all the output responses with respect to the design parameters, an objective function has been defined and a desirability function based approach has been implemented. Finally, the optimized values of the geometric design parameters and corresponding output responses are verified through FEM simulations. If the predicted values from the optimization results are not within 95% confidence interval, then it is recommended to return to the step of generating sample data space with more data points using LHS to obtain a more accurate response surface.

### 3.2. Design Parameters and Their Levels

Table 1 shows the design parameters with respective low and high levels considered for the DACE based optimization of MEMS accelerometer. The design parameters are coded as $X_{I}$ for i=1, 2,…,8. The low and high levels of the design parameters are based on the geometric configuration of design, operating environmental conditions, and microfabrication process constraints. Figure 1 shows the dimensions of the T-shape spring in terms of lengths and width and represents the design parameters $X_{2},{\;X}_{3}$, and $X_{4}$. For the design parameter $X_{4}$, the minimum width that can be set for a beam having a length greater than 100 μm is 6 μm as per the SOIMUMPs process constraint. The proposed MEMS accelerometer is to be optimized for measuring the input acceleration in the range of ±1 g to ±25 g.

The MEMS accelerometers for most of the applications, especially in space applications are required to operate in the temperature range of −40 °C (233.15 K) to 100 °C (373.15 K). Therefore, the low and high levels for the design parameter operating temperature ($X_{6}$) are kept in this range. The range for design parameter operating pressure ($X_{7}$) is kept 100 Torr (sub-atmospheric pressure) for a low level to 760 Torr (atmospheric pressure) for a high level. This range is selected to analyze the dependency of noise and other output responses of the MEMS accelerometer on the operating pressure. The last design parameter $X_{8}$ is the frequency ratio (FR), which is the ratio of any frequency value (lower than natural frequency) to the obtained natural frequency of the specific design run.

### 3.3. Latin Hypercube Sampling (LHS) Based Space Filling Design

The selection of design space for computer experiments is based on two basic principles: (a) any combination of design parameters should not appear more than once in the design space since replication is only required in physical experiments to account for random errors and (b) the different combinations of design parameters should cover the whole region of the design space, so that different behaviors of output responses in different areas of the design space can be analyzed [39]. This is also because due to the complex nature of computer simulations, the response behavior may change across the design space. The space-filling designs are generally used for DACE due to the evenly spread of design points throughout the design space. Among various types of space-filling designs, the Latin hypercube sampling (LHS) approach is most widely used. The LHS creates the design space for design parameters by maximizing the minimum distance between any two design points and the design points evenly cover the whole design space. This ensures enough degree of freedom to estimate both the linear and quadratic effects of design parameters and minimize the discrepancy between the observed values. For the LHS and other space-filling design, it is recommended that the number of simulation runs must be at least 10X, where X is the number of design parameters [26]. Table S1 shows the LHS based design matrix for the design parameters considered for the optimization of the MEMS accelerometer.

### 3.4. FEM Modelling of 2-DoF MEMS Accelerometer

**Figure 3:**
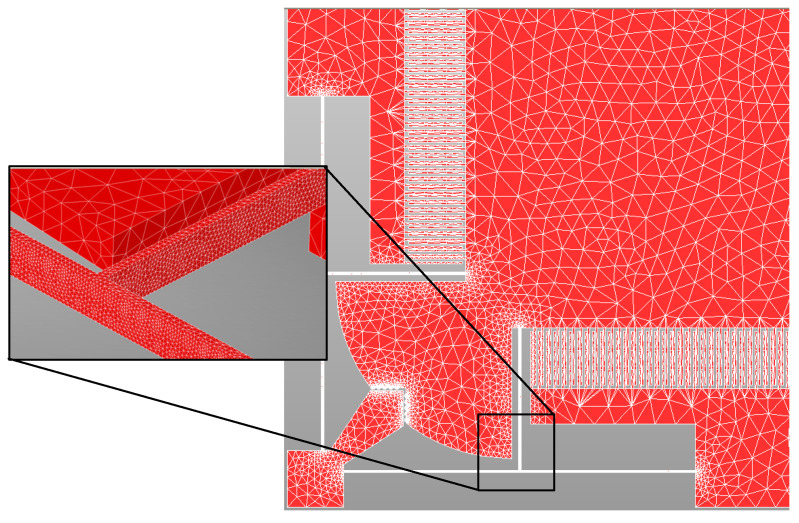
3D meshed model of the MEMS accelerometer.

The output responses, corresponding to the LHS based design matrix, are obtained through FEM simulations in CoventorWare^®®^ software. The MEMS accelerometer is meshed using three-dimensional (3D) solid tetrahedral 739,599 elements with a total number of 272,580 elements for the central proof mass and elements for the mechanical suspension beams. A fine mesh is used for the mechanical suspension beams, with multiple elements along the thickness, as shown in Figure 3. The material properties of thin film Silicon are included as input in the FEM analysis with Young’s Modulus of 169 GPa, Poisson ratio of 0.29, and density of 2300 kg/m^3^ [36].

The natural frequency of the MEMS accelerometer is obtained through modal analysis in the CoventorWare MemMech module. The proof mass displacement, corresponding to an input acceleration, is obtained for each simulation run using harmonic analysis and considering the air damping effect. In the dual-axis MEMS accelerometer design, both the squeeze and slide air film damping result in energy dissipation. For an input acceleration, the squeeze film air damping occurs between the stator and rotor combs in the active sense axis, and a slide film air damping occurs between the two sets of combs in the inactive axis. In the present work, for each simulation run, the value of air damping is first computed by using the CoventorWare DampingMM module and subsequently used in the harmonic analysis. In the FEM analysis, the gas rarefaction effects are considered for each simulation run since the dominant airflow regime is slip flow for an air gap of 2.5 μm between the stator and rotor combs of the MEMS accelerometer. For each simulation run in the design matrix, the computed values of squeeze number and Reynolds number are in the range of 10^−3^ thus air compressibility and inertial damping effects are ignored. A detailed description of modelling of thin film air damping effects in capacitive sensing combs is given by the authors in [40,41].

For a bias voltage of 2.25 V to the parallel capacitive sensing plates, coupled field electric-structural analysis is performed to find the value of pull-in voltage. The capacitance change for each simulation run is obtained through the CoventorWare MEMS+ module. Based on the FEM analysis based on natural frequency and quality factor, the value of BNEA for each simulation run, at a given operating temperature, is obtained by using the following relation [42].

(5) $$\mathrm{BNEA}=\sqrt{\frac{4K_{B}T\omega_{n}}{m_{p}Q}}$$

where $K_{B}$ is the Boltzmann constant, T is the operating temperature, $\omega_{n}$ is the natural frequency, $m_{p}$ is the value of proof mass, and Q is the quality factor.

## 4. Development of GP Based Metamodels for the Output Responses

For the LHS based design matrix, with corresponding output responses obtained through FEM simulations (Table S1), the Gaussian process model is fitted to obtain the metamodels for the output responses. For each output response, the value of hyperparameter θ is obtained for each design parameter by minimizing the negative log-likelihood and the values are reported in Table 2. These values are used in Equation (3) to control the length scale and smoothness of modelled Gaussian surface for each output response. After fitting the Gaussian process model, the predictive values for the output response are obtained based on Equation (4) and interactions between design parameters for each output response are also estimated. Table 3 lists the values for interaction between significant design parameters for each output response.

### 4.1. Significant Design Parameters and Interaction Analysis for Natural Frequency ($Y_{1}$)

**Figure 4:**
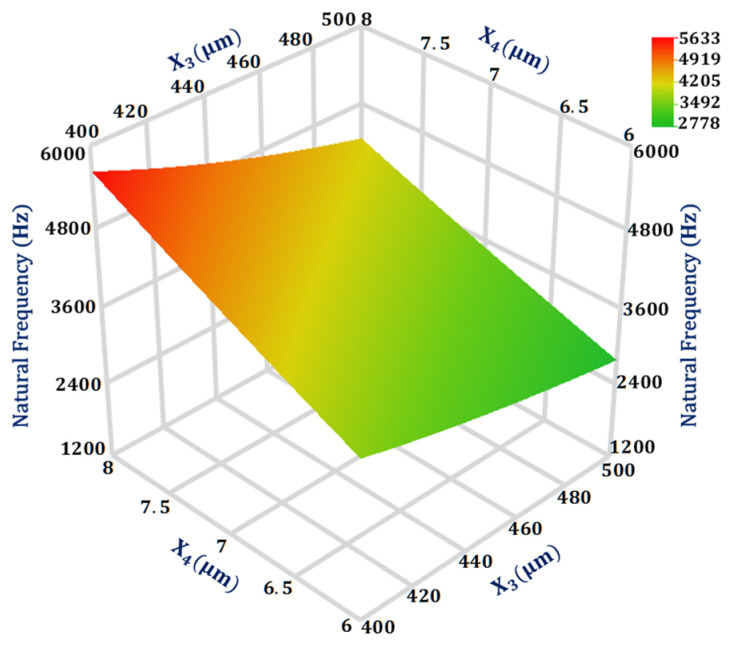
Response surface plot for natural frequency as a function of design parameters X_3_ and X_4_.

From the Gaussian process model analysis, the most significant interaction is between the design parameters $X_{3}$ and $X_{4}$ for the natural frequency with a value of 0.005, as given in Table 3. Figure 4 shows the 3D response surface plot for the natural frequency of the MEMS accelerometer with respect to design parameters $X_{3}$ and $X_{4}$. The response surface plot is obtained using GP based metamodel and the value of all other design parameters are kept at their mean value. The result shows that the natural frequency is more sensitive to the change in $X_{4}$ in comparison to $X_{3}$. Moreover, with $X_{4}$= 8 μm the natural frequency is much more sensitive to change in the $X_{3}$ value in comparison to when $X_{4}$= 6 μm.

### 4.2. Significant Design Parameters and Interaction Analysis for Proof Mass Displacement ($Y_{2}$)

**Figure 5:**
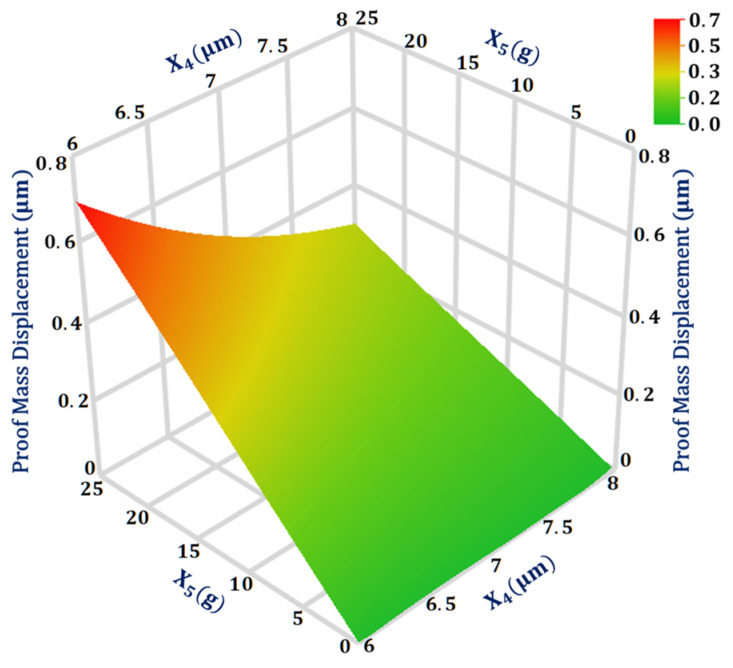
Response surface plot for proof mass displacement as a function of design parameters X_4_ and X_5_.

The maximum interaction value is between the two design parameters $X_{4}$ and $X_{5}$ for the output response proof mass displacement. Figure 5 shows the 3D surface plot for the proof mass displacement with respect to the design parameters $X_{4}$ and $X_{5}$ with all other design parameters at their mean value. The plot shows that for input acceleration above 20 g, the proof mass displacement becomes more sensitive to the change in the suspension beam width. Moreover, the proof mass displacement shows a linear increase in the value with the increase in the input acceleration.

### 4.3. Significant Design Parameters and Interaction Analysis for Pull-In Voltage ($Y_{3}$)

**Figure 6:**
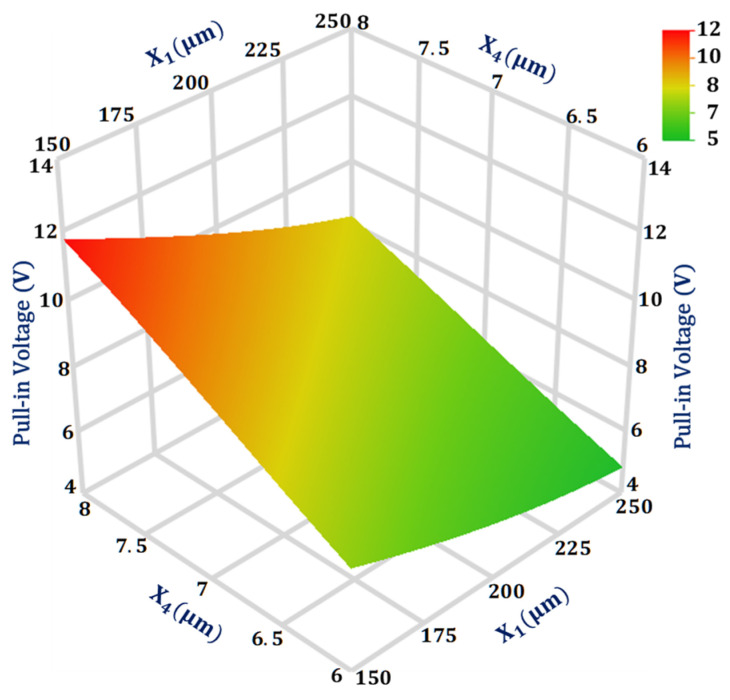
Response surface plot for pull-in voltage as a function of design parameters X_1_ and X_4_.

For the output response pull-in voltage, the design parameters interaction value is highest between $X_{1}$ and $X_{4}$. Figure 6 shows the 3D surface plot for the interaction analysis between these two design parameters on the pull-in voltage value. The plot shows that the pull-in voltage is more sensitive to the change in design parameter $X_{4}$ in comparison to $X_{1}$. Moreover, the effect of change in $X_{4}$ value from 6 to 8 μm has less effect on the pull-in voltage value when $X_{1}$ is equal to 250 μm in comparison to when $X_{1}$ value is 150 μm.

### 4.4. Significant Design Parameters and Interaction Analysis for Capacitance Change ($Y_{4}$)

**Figure 7:**
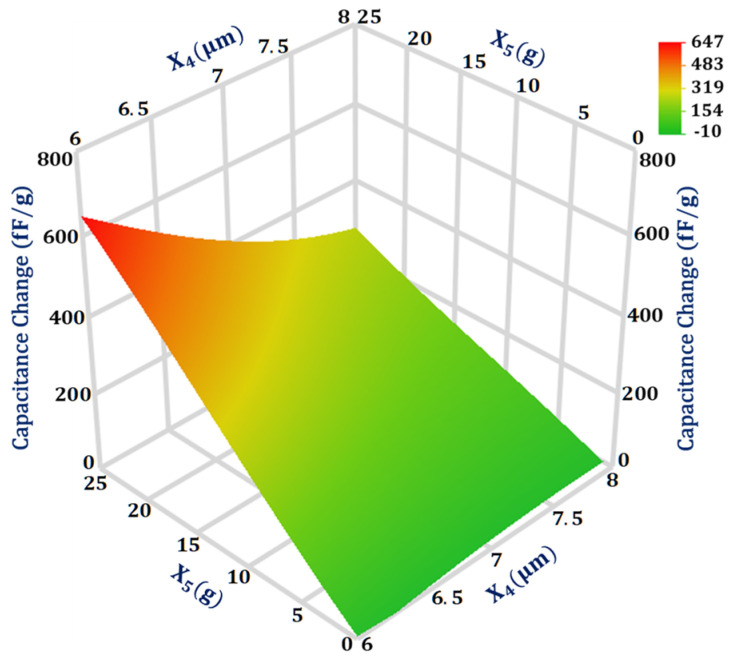
Response surface plot for capacitance change as a function of design parameters X_4_ and X_5_.

As per the GP model analysis, the interaction between two design parameters $X_{4}$ and $X_{5}$ for the capacitance change has a maximum magnitude with a value of 0.038. The 3D surface plot in Figure 7 shows that when $X_{4}$ is at a high level of 8 μm the effect of change in $X_{5}$ on capacitance change is less in comparison to when $X_{4}$ is at a low level of 6 μm. Moreover, the effect of change in $X_{4}$ on the capacitance change is more when $X_{5}$ is at a high value of 25 g in comparison to its low value of 1 g.

### 4.5. Significant Design Parameters and Interaction Analysis for BNEA ($Y_{5}$)

**Figure 8:**
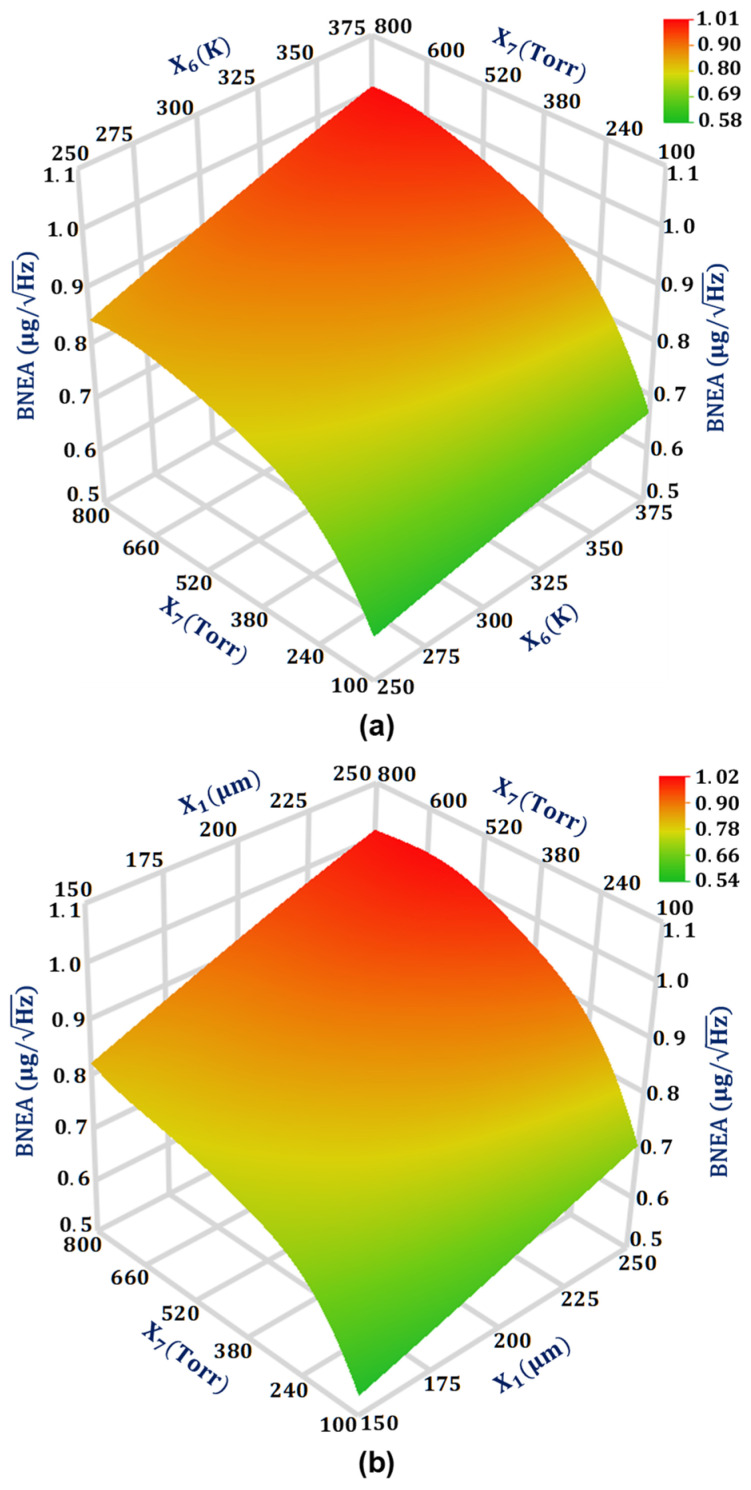
Response surface plot for BNEA: (**a**) as a function of design parameters X_6_ and X_7_, and (**b**) as a function of design parameters X_1_ and X_7_.

For the output response BNEA, the design parameters that have a significant effect on the output response BNEA are $X_{1}$, $X_{6}$, and $X_{7}$, respectively. The two design parameter interactions $X_{1}X_{7}$ and $X_{6}X_{7}$ are comparable to each other with values 0.007285 and 0.007235, respectively, for the output response BNEA. Figure 8a shows the interaction plot of design parameters $X_{1}$ and $X_{7}$ for the BNEA. The results show that BNEA increases linearly with an increase in the $X_{1}$ from 150 μm to 250 μm. This can be attributed to the fact that with the increase in the overlap value the air damping increases which subsequently decreases the quality factor. From Equation (5), it is clear that a decrease in the quality factor leads to an increase in the BNEA value. The effect of an increase in $X_{7}$ from a low value of 100 Torr to the atmospheric air pressure of 760 Torr has a highly non-linear effect on the BNEA value for the MEMS accelerometer. Figure 8b shows the interaction plot of the design parameters $X_{6}$ and $X_{7}$ for the BNEA. The results show that the BNEA increases linearly with an increase in the design parameter $X_{6}$ value from 250 K to 375 K.

### 4.6. Prediction Accuracy of the Fitted GP Metamodels

The prediction accuracy of the developed GP based metamodels for the output responses is analyzed by estimating the mean absolute error (MAE), root mean square error (RMSE), and correlation coefficient I for each response. The MAE, RMSE, and R for an output response can be calculated as follows [43,44]:

(6) $$\mathrm{MAE}=\frac{1}{k}\overset{k}{\underset{i=1}{\sum }}\left|y_{\mathrm{oi}}-y_{\mathrm{pi}}\right|$$

(7) $$\mathrm{RMSE}=\sqrt{\frac{1}{k}\overset{k}{\underset{i=1}{\sum }}{\left(y_{\mathrm{oi}}-y_{\mathrm{pi}}\right)}^{2}}$$

(8) $$R=\frac{k\sum_{i=1}^{k}y_{\mathrm{oi}}\times y_{\mathrm{pi}}-\sum_{i=1}^{k}y_{\mathrm{oi}}\sum_{i=1}^{k}y_{\mathrm{pi}}}{\sqrt{\left[k\sum_{i=1}^{k}y_{\mathrm{oi}}{}^{2}-{\left(\sum_{i=1}^{k}y_{\mathrm{oi}}\right)}^{2}\right]\times \left[k\sum_{i=1}^{k}y_{\mathrm{pi}}{}^{2}-{\left(\sum_{i=1}^{k}y_{\mathrm{pi}}\right)}^{2}\right]}}$$

where $y_{\mathrm{oi}}$ is the actual observed value for an output response for the *i*th run of the simulation design matrix and $y_{\mathrm{pi}}$ is the predicted value of output response for the *i*th run. The MAE gives an estimation of the mean of the absolute errors for each simulation run, whereas RMSE gives a measure of the standard deviation of the residuals and depicts that how far the predicted points are from the fitted linear regression line. The smaller the MAE and RMSE values are, the better the points fit actual data values. The R-value close to one indicates that the linear relationship between the observed and the predicted values is positive, and the points lie nearly along a fit line. Table 4 lists the values of MAE, RMSE, and R for the output responses. These values suggest that the developed metamodels for the output responses are accurate in predicting the output responses.

## 5. Multi-Response Optimization

### 5.1. Optimization Objective Function

The first step in the multi-response optimization of the proposed MEMS accelerometer design is to define the desired optimization objective function, for a given set of constraints for the design parameters. The objective function for the MEMS accelerometer is given as follows:

(9) $$\begin{array}{l} \mathrm{Minimize}\text{—}\mathrm{Natural}\;\mathrm{frequency}\;(Y_{1})\\ \mathrm{Maximize}\text{—}\mathrm{Proof}\;\mathrm{mass}\;\mathrm{displacement}\;(Y_{2})\\ \mathrm{Maximize}\text{—}\mathrm{Pull}\text{-}\mathrm{in}\;\mathrm{voltage}\;(Y_{3})\\ \mathrm{Maximize}\text{—}\mathrm{Capacitance}\;\mathrm{change}\;(Y_{4})\\ \mathrm{Minimize}\text{—}\mathrm{BNEA}\;(Y_{5})\\ \mathrm{such}\;\mathrm{that}\\ 150\;µm\leq X_{1}\leq 250\;µm\\ 400\;µm\leq X_{2}\leq 500\;µm\\ 400\;µm\leq X_{3}\leq 500\;µm\\ 6\;µm\leq X_{4}\leq 8\;µm\\ X_{5}=25\;g\\ X_{6}=300\;K\\ X_{7}=760\;\mathrm{Torr}\\ 0.1\leq X_{8}\leq 0.5\; \end{array}$$

The value of the design parameter $X_{5}$ is set to a maximum input acceleration of 25 g. Similarly, the design parameters $X_{6}$ and $X_{7}$ are set to the atmospheric air pressure and room temperature of 760 Torr and 300 K, respectively.

### 5.2. Desirability Function Based Simultaneous Multi-Response Optimization

Various techniques have been reported in the literature to simultaneously optimize multiple output responses, including the distance function approach [45], loss function approach [46], and desirability function approach [47]. The desirability function approach was initially proposed by Harrington [48] in the form of exponential functions and later modified by Derringer and Suich [47] and Del Castillo et al. [49]. In this approach, the estimated value for each output response is transformed to a scale free value $(d_{i}{(y}_{i}\left(x))\right)$ which is termed as desirability. The $d_{i}{(y}_{i}(x$)) value is scaled to be between 0 and 1 with 0 and 1 being the least and most optimal values, respectively. The overall desirability function is defined by taking the geometric mean of the individual desirability value for each output response. The desirability functions proposed by Derringer and Suich [47] contain non-differentiable target points and hence only search methods can be used for optimization. Later, Del Castillo et al. [49] proposed alternative piece-wise continuous desirability functions that account for non-differentiable points, and hence more efficient gradient-based algorithms can be applied to obtain the optimal solution. The non-differentiable desirability function for an output response is given as follows:

(10) $$d_{i}\left(y_{i}\left(x\right)\right)=\{\begin{array}{cc} a_{0}+b_{0}y_{i}\left(x\right) & \mathrm{if}\;L<y_{i}\left(x\right)\leq T-\delta \\ f\left(y_{i}\left(x\right)\right) & \mathrm{if}\;T-\delta \leq y_{i}\left(x\right)\leq T+\delta \\ a_{1}+b_{1}y_{i}\left(x\right) & \mathrm{if}\;T+\delta \leq y_{i}\left(x\right)\leq U\\ 0 & \mathrm{otherwise} \end{array}$$

where L, U, and T represent the lower, upper, and target values for an output response, respectively. The term δ=(U−L)/50 defines the small range around the non-differentiable point. The terms $a_{0}$, $a_{1}$, $b_{0}$, and $b_{1}$ are constants and a detailed discussion to find the values of these constants are given in Del Castillo et al. [49]. The function $f\left(y_{i}\left(x\right)\right)$ is the polynomial approximation function that corrects for the non-differentiable points and is given as follows:

(11) $$f\left(y_{i}\left(x\right)\right)=A+{\mathrm{By}}_{i}\left(x\right)+{\mathrm{Cy}}_{i}{\left(x\right)}^{2}+{\mathrm{Dy}}_{i}{\left(x\right)}^{3}+{\mathrm{Ey}}_{i}{\left(x\right)}^{4}$$

A, B, C, D, and E are constant parameters. For simultaneous optimization of all the responses, a function named as global desirability is defined by taking the geometric mean of all the individual desirability values for the output responses and is given as follows:

(12) D=(∏i=1kdizi)1∑ zi=(d1z1×d2z2×d3z3×…×dkzk)1∑ zi

where k is the number of output responses to be optimized and $z_{i}\;\left(0\;<\;z_{i}\;<1\right)$ reflects the importance of each output response relative to others. The importance value ($z_{i}$) for each output response is scaled so that they sum up to one for all the responses.

**Figure 9:**
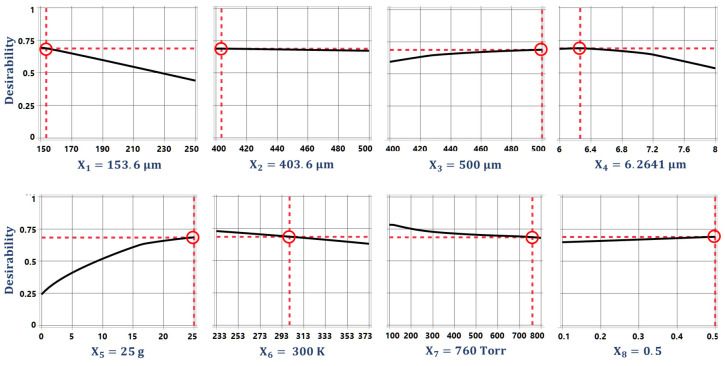
The optimized values of design parameters along with their individual desirability.

**Figure 10:**
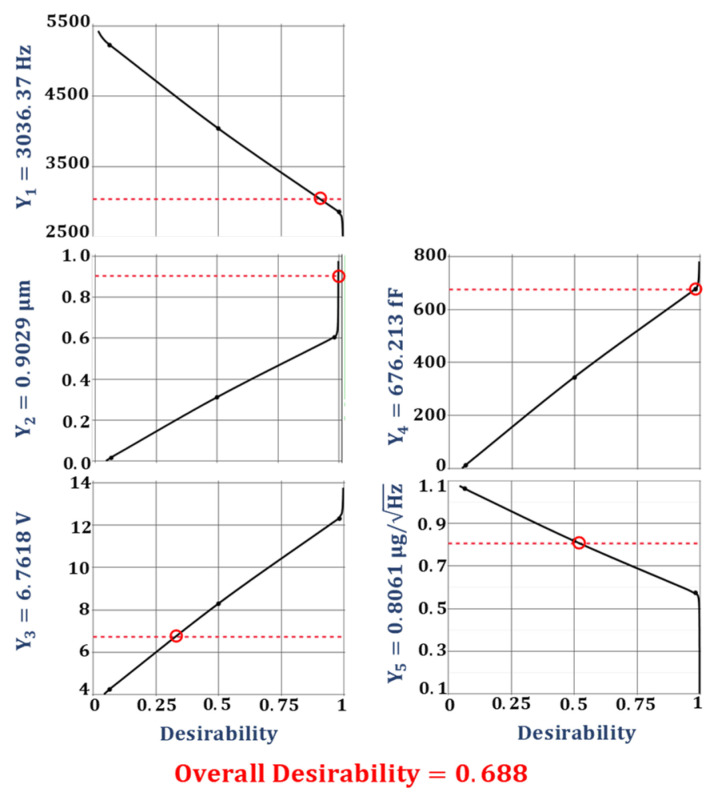
The predicted values of the output responses along with their individual desirability.

The optimization of the desirability function is generally carried out using either search or gradient-based algorithms [47,49]. The search algorithm-based optimization is a derivative-free approach and can be applied for the optimization of desirability functions whose derivative does not exist. The search algorithm-based optimization requires the functions to have continuous first derivatives and is a more efficient and widely used method. The multi-response optimization of the desired objective function for the proposed 2-DoF MEMS accelerometer is performed using a gradient descent algorithm. Figure 9 shows the optimal values for the design parameters along with their individual desirability values. Figure 10 shows the individual desirability values for the output responses and corresponding optimized predicted values for each response. The maximum value of overall desirability obtained is 0.688 with a predicted value of 3037.37 Hz for the natural frequency, 0.9029 µm for the proof mass displacement, 6.7618 V for the pull-in voltage, 676.213 fF for the capacitance change, and 0.8061 $\mu g/\sqrt{\mathrm{Hz}}$ for the BNEA.

### 5.3. Verification of Predicted Values for the Output Responses

The desirability function based predicted values for the output responses are further verified through FEM simulations to validate both the metamodel and desirability function based optimization approach for the proposed MEMS accelerometer design. The MEMS accelerometer is modelled by using the optimized geometric design parameter values shown in Figure 9.

#### 5.3.1. Natural Frequency Analysis

**Figure 11:**
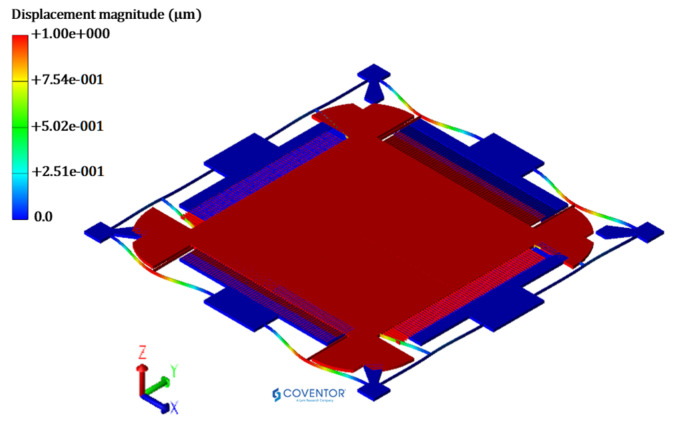
Eigen mode shape for MEMS accelerometer having 1st mode along Y-direction at 3038.133 Hz.

The predicted optimized value of the natural frequency is verified through FEM based simulation in the CoventorWare MemMech module. Figure 11 shows the natural frequency of the MEMS accelerometer with the corresponding mode shape. The natural frequency value obtained through simulation is 3038.1 Hz, which lies within the 95% confidence prediction interval i.e., 2980 Hz ≤ $Y_{1}$ = 3038.1 Hz ≤ 3090 Hz.

#### 5.3.2. Frequency Response Analysis

**Figure 12:**
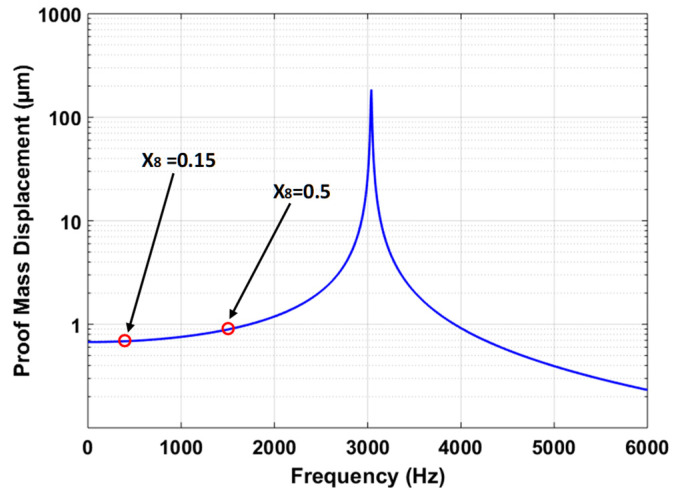
Frequency response of the MEMS accelerometer for an input acceleration 25 g.

The predicted value of the output response proof mass displacement is verified by using the harmonic analysis in the CoventorWare MEMS+ module. Figure 12 shows the frequency response curve for the MEMS accelerometer at an input acceleration of 25 g. The results show that at frequency ratio ($X_{8}$) of 0.5, the displacement amplitude of the proof mass is 0.898 µm. lies within the 95% prediction confidence prediction interval, i.e., 0.891 µm ≤ $Y_{2}$ = 0.898 µm ≤ 0.915 µm. The $X_{8}$ = 0.5 corresponds to an operational bandwidth of 1519 Hz. However, the MEMS accelerometers are generally designed for operational bandwidth of 0–450 Hz. Figure 12 shows that for $X_{8}$ = 0.15 with operational bandwidth of 0–450 Hz, the proof mass displacement amplitude is relatively linear with a maximum value of 0.688 µm for the proof mass displacement.

#### 5.3.3. Pull-In Voltage Analysis

**Figure 13:**
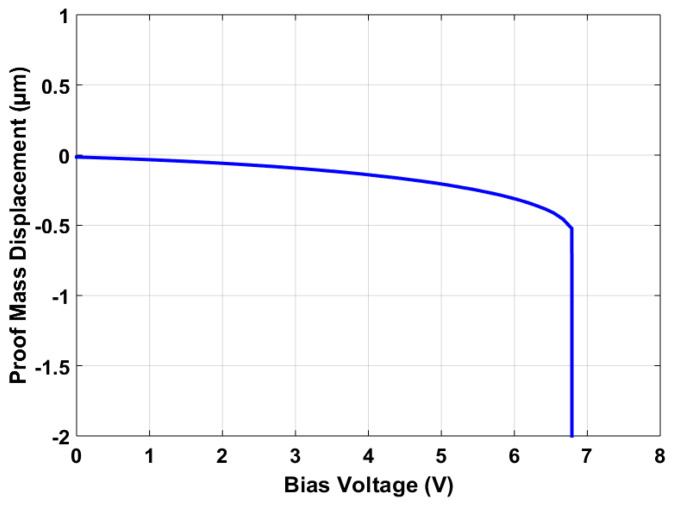
Proof mass displacement vs. applied bias voltage graph for the optimized MEMS accelerometer design.

Figure 13 shows the bias voltage vs. proof mass displacement graph for the final optimized MEMS accelerometer design. The results show that the maximum bias voltage that can be applied to the stator and rotor sensing combs must be less than the maximum pull-in value of 6.79 V. This pull-in voltage value is in close agreement with a predicted value of 6.76 V and lies within the 95% confidence prediction interval, i.e., 6.36 V ≤ $Y_{3}$ = 6.79 V ≤ 7.16 V.

#### 5.3.4. Capacitance Change Analysis

**Figure 14:**
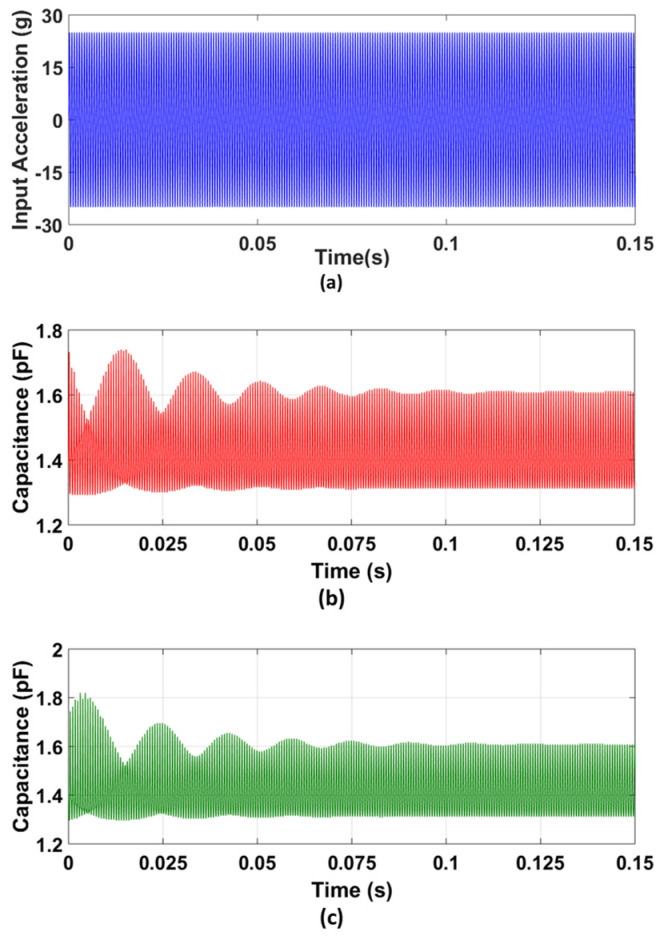
Capacitance change analysis for the optimized MEMS accelerometer: (**a**) input acceleration at X_8_, (**b**) capacitance change in the small gap, and (**c**) capacitance change in the large gap between the rotor and stator sensing combs displacement vs. applied bias voltage graph for the optimized MEMS accelerometer design.

For the verification of the predicted capacitance change for the final optimized MEMS accelerometer design, the accelerometer model developed in the CoventorWare MEMS+ module is interfaced with MATLAB Simulink. A bias voltage of 2.25 V is applied between the proof mass and stator electrodes, since the proposed accelerometer is designed to be interfaced with the commercial capacitive to voltage conversion readout IC, MS3110 [50]. An input acceleration of 25 g at $X_{8}$ = 0.5, i.e., 1519 Hz is given as an input to the MEMS accelerometer, as shown in Figure 14a. Figure 14b,c show the corresponding gap and anti-gap capacitance. The net change in capacitance is 694 fF which is close to the predicted value of 676.2 fF and lies within the 95% confidence prediction interval, i.e., 618 fF ≤ $Y_{4}$ = 694 fF ≤ 735 fF. For $X_{8}$ = 0.15, i.e., an input acceleration frequency of 450 Hz, the net change in capacitance for the MEMS accelerometer is 523 fF.

#### 5.3.5. Estimation of Brownian Noise Equivalent Acceleration (BNEA)

For the estimation of BNEA, the MEMS 2-DoF accelerometer with optimal parameters is first subjected to damping analysis in the CoventorWare DampingMM module to obtain squeeze and slide film damping coefficients. The operating air pressure and temperature are set at room conditions, i.e., 300 K and 760 Torr. These coefficients are then used to estimate the value of BNEA based on Equation (5). The value of BNEA is estimated to be 0.805 μg/$\sqrt{\mathrm{Hz}}$ which is close to the predicted value of 0.8061 μg/$\sqrt{\mathrm{Hz}}$ and lies within the 95% confidence interval, i.e., 0.797 μg/$\sqrt{\mathrm{Hz}}$ ≤ $Y_{5}$ = 0.805 μg/$\sqrt{\mathrm{Hz}}$ ≤ 0.815 μg/$\sqrt{\mathrm{Hz}}$.

## 6. Discussion

The capacitive MEMS accelerometers are multiphysics sensors involving complex interactions between the electrical–mechanical–fluidic domains. Table 5 shows a comparison of different capacitive MEMS accelerometer design and analysis approaches presented in the literature. Most of the MEMS accelerometer design analysis and optimization approaches involve the traditional varying one or two design factors at a time and observing its effect on a specific output response. In most cases, the design analysis is only focused on the geometric design parameters. However, the performance of capacitive MEMS accelerometers is strongly dependent on the sensor operating conditions including air pressure and temperature. The MEMS accelerometer operating temperature and air pressure have a strong influence on the air damping and hence on the dynamic response of these sensors. The design analysis and optimization of multiphysics MEMS devices in general and capacitive MEMS accelerometers, in particular, requires analyzing the output responses related to different physics domains simultaneously with respect to both geometric design parameters and device operating conditions. 

**Figure 15:**
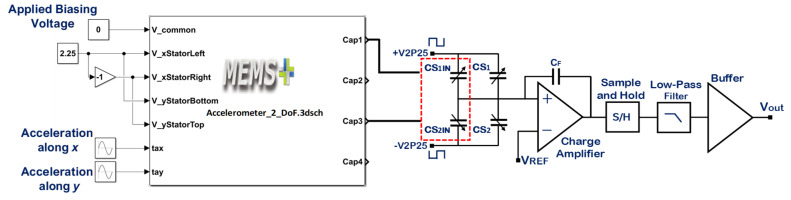
Integration of the optimized MEMS accelerometer behavioral model with the C/V converter readout electronics.

**Figure 16:**
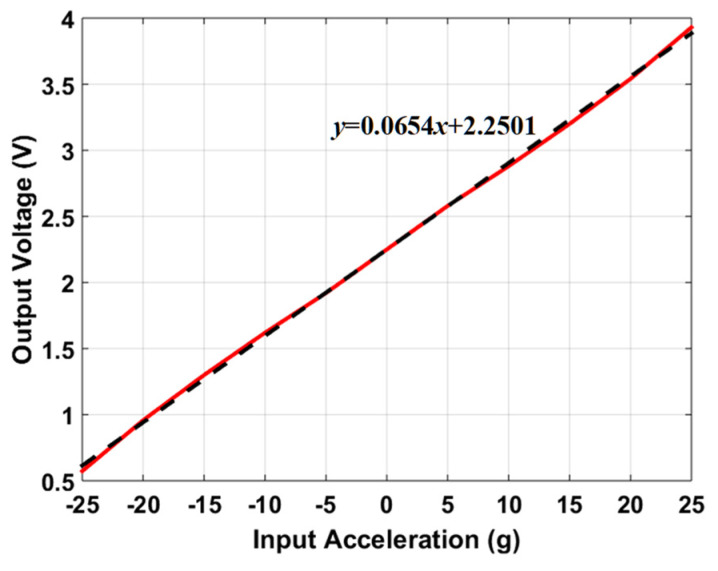
The output voltage of the readout electronics for an input acceleration in the range of −25 g to +25 g along the X-axis.

One of the important performance parameters for MEMS accelerometers is voltage sensitivity which is dependent on the capacitive to voltage readout electronics to estimate the voltage sensitivity of the optimized MEMS accelerometer, the behavioral model of the accelerometer is interfaced with the commercially available capacitance to voltage convertor Universal Readout IC^TM^ MS3110 [50] in MATLAB Simulink environment. This readout circuitry is capable of measuring both single and differential capacitance with a resolution of $4\;\mathrm{aF}/\sqrt{\mathrm{Hz}}$. Figure 15 illustrates the integration of 2-DoF MEMS accelerometer behavioral model with MS3110. The working principle of the IC for the measurement of differential capacitance is based on charge amplification, followed by sample and hold, a low-pass frequency filter, and a buffer amplifier. A bias voltage of 2.25 V is applied between the proof mass and stator electrodes of the MEMS accelerometer. For an input acceleration along X-axis, the capacitance mismatch between the input capacitors, the difference ${\mathrm{CS}}_{1}-{\mathrm{CS}}_{2}$ is zero and ΔC will be equal to the difference between the capacitors Cap1 and Cap3 as obtained in the capacitance change analysis section. The feedback capacitor $C_{F}$ value is adjusted to 3.2 pF to keep the output voltage in the range of 0.5 to 4 V. Figure 16 shows the effect of change in input acceleration on the output voltage of the readout interface circuit. The graph shows that the maximum and minimum values for output voltage are 3.93 and 0.573 V respectively with a voltage sensitivity of 65.4 mV/g for the final optimized MEMS accelerometer design.

## 7. Conclusions

In this paper, a simulation-based design optimization approach for the MEMS accelerometer is presented by using DACE based design space sampling and development of metamodels for output responses of interest using GP modelling. The DACE based LHS sampling allows to cover the whole design space of the MEMS accelerometer by using only 10X simulation runs, where X = 8 is the number of design parameters. Moreover, combined DACE based sampling and GP modelling allows to analyze the interaction between the design parameters and their effect on the output responses simultaneously instead of conventional varying one factor at a time based optimization approach. The output responses of the MEMS accelerometer including natural frequency, proof mass displacement, pull-in voltage, capacitance change, and BNEA are obtained using FEM and behavioral model simulations for each simulation run in the LHS sampling design matrix. The Gaussian process regression is used for developing the metamodel for each output response which allowed to analyze the effect of design parameters and their interaction on the output responses in detail. The accuracy of the developed metamodels for predicting the output responses is proved by estimating the statistical parameters. An optimization objective function for the MEMS accelerometer is defined, and simultaneous optimization of the output responses is carried out using a combined desirability function and gradient-based algorithm. The predicted optimal values of the output responses are verified through FEM simulation and the obtained values showed a close agreement with the prediction. The optimized values of output responses for the MEMS accelerometer are natural frequency of 3036.37 Hz, proof mass displacement of 0.903 μm at ±25 g, pull-in voltage value of 6.762 V, output capacitance change of 676.213 fF at ±25 g, and BNEA of 0.8061 μg/$\sqrt{\mathrm{Hz}}$. Based on the optimized values of the proof mass displacement and capacitance change and for an operational bandwidth of 0–450 Hz, the mechanical, capacitance, and voltage sensitivity of the MEMS accelerometer are 0.027 μm/g, 22.9 fF/g, and 65.4 mV/g, respectively. The DACE based optimization technique for the multi-response optimization of MEMS accelerometer using Gaussian process regression overcomes the limitations of traditional DoE based optimization and can be implemented for multiphysics MEMS devices with complex geometric configurations. In the future, the application of DACE based optimization methodology considering the microfabrication process uncertainties and size effects can be investigated for the reliability-based optimization of the MEMS accelerometers. 

## Figures and Tables

**Table 1:** Design parameters for the MEMS accelerometer design optimization.

Code	Design Parameters	Low Level	High Level
X_1_	Overlap length of comb	150 µm	250 µm
X_2_	Length of suspension beam 1	400 µm	500 µm
X_3_	Length of suspension beam 2	400 µm	500 µm
X_4_	Width of suspension beam	6 µm	8 µm
X_5_	Input acceleration	1 g	25 g
X_6_	Operating temperature	233.15 K	373.15 K
X_7_	Operating pressure	100 Torr	760 Torr
X_8_	Frequency ratio	0.1	0.5

**Table 2:** Obtained values for hyperparameter θ after fitting the Gaussian process model.

Output Responses	Design Parameters							
	X_1_	X_2_	X_3_	X_4_	X_5_	X_6_	X_7_	X_8_
Natural frequency (Y_1_)	4.23 × 10^−6^	5.52 × 10^−6^	9.74 × 10^−5^	0.36	0	0	0	0
Proof mass displacement (Y_2_)	0	0	8.26 × 10^−5^	0.34	0.01	0	0	1.24
Pull-in voltage (Y_3_)	6.29 × 10^−5^	1.23 × 10^−5^	5.49 × 10^−5^	0.14	1.49 × 10^−5^	4.29 × 10^−6^	6.18 × 10^−9^	2.19
Capacitance change (Y_4_)	2.19 × 10^−5^	1.01 × 10^−6^	0.000026	0.1235	0.0018	7.66 × 10^−7^	3.82 × 10^−8^	0.88
BNEA (Y_5_)	0.000142	0	0	0	0	5.26 × 10^−5^	6.03 × 10^−6^	0

**Table 3:** Significant design parameters interactions for the output responses.

Output Responses	Significant Design Parameters Interaction	
	Design Parameters	Interaction Value
Natural frequency (Y_1_)	$X_{3}X_{4}$	0.005
Proof mass displacement (Y_2_)	$X_{4}X_{5}$	0.031
Pull-in voltage (Y_3_)	$X_{1}X_{4}$	0.004
Capacitance change (Y_4_)	$X_{4}X_{5}$	0.038
BNEA (Y_5_)	$X_{1}X_{7}$, $X_{6}X_{7}$	0.007, 0.007

**Table 4:** Prediction accuracy estimates for the fitted GP metamodels.

Output Responses	MAE	RMSE	R
Natural frequency (Y_1_)	29.64 Hz	41.19 Hz	0.998
Proof mass displacement (Y_2_)	0.024 μm	0.034 μm	0.981
Pull-in voltage (Y_3_)	0.085 V	0.134 V	0.997
Capacitance change (Y_4_)	10.178 fF	14.05 fF	0.996
BNEA (Y_5_)	$0.019\;\mu g/\sqrt{\mathrm{Hz}}$	$0.029\;\mu g/\sqrt{\mathrm{Hz}}$	0.973

**Table 5:** Comparison of the proposed optimization methodology for capacitive MEMS accelerometers with that presented in the literature.

Reference	Optimization Approach	Design Factor(s)	Output Response(s)	Simultaneous Optimization of Output Responses
Mohammed et al. [13]	One design factor	Geometric parameters	Differential capacitance	No
Keshavarzi and Hasani [20]	Two design factors	Geometric parameters	Capacitance sensitivity	No
Ramakrishnan et al. [21]	Traditional DOE	Geometric parameters	Mechanical displacement, stress, bandwidth	No
Li et al. [22]	One design factor	Geometric parameters	Capacitance sensitivity	No
Shi et al. [51]	Two design factors	Geometric parameters	Mechanical sensitivity, stress, natural frequency	No
Martha et al. [52]	Two design factors	Geometric parameters	Pull-in voltage, capacitance sensitivity	No
This work	Combined DACE and GP Modelling	Geometric parameters, Operating conditions	Natural frequency, mechanical displacement, capacitance sensitivity, BNEA, pull-in voltage	Yes

## Supplementary Materials

The following are available online at https://www.mdpi.com/article/10.3390/s21217242/s1, Table S1: The sets of design parameters for computer experiments on MEMS 2-DoF accelerometer design optimization.
