# Supplementary material for: A Systematic Design Optimization Approach for Multiphysics MEMS Devices Based on Combined Computer Experiments and Gaussian Process Modelling
Source: Sensors (Basel). 2021 Oct 30;21(21):7242. doi: 10.3390/s21217242 (PMC8587333; doi:10.3390/s21217242)
Supplement: Supplementary file 1 [file sensors-21-07242-s001.zip › sensors-1370782-supplementary.pdf]

Table S1: LHS based design matrix for the eight design parameters and corresponding five output responses obtained through FEM simulations

Run #	Input Factors								Output Responses				
	X_1	X_2	X_3	X_4	X_5	X_6	X_7	X_8	Y_1	Y_2	Y_3	Y_4	Y_5
1	243.67	431.65	443.04	6.1519	24.0886	250.87	651.39	0.2519	3339.049	0.5869	5.2525	676	0.9224
2	165.19	436.71	484.81	6.2532	11.0253	351.88	492.66	0.4494	3089.005	0.3678	6.4647	297	0.8563
3	153.8	430.38	406.33	7.519	22.5696	296.95	484.3	0.4038	5134.294	0.2561	12.3232	238	0.7657
4	171.52	440.51	403.8	6.1772	10.4177	265.05	459.24	0.1911	3834.470	0.1850	8.0808	145	0.7643
5	229.75	462.03	412.66	7.5443	5.2532	348.34	216.96	0.2367	4941.468	0.0572	9.0452	58	0.8650
6	213.29	411.39	418.99	7.3671	24.3924	355.43	233.67	0.2114	4760.820	0.2830	9.2462	275	0.8547
7	170.25	470.89	473.42	6.9367	1.3038	328.85	200.25	0.3025	3666.030	0.0269	7.8788	21	0.7145
8	176.58	449.37	415.19	7.6203	20.7468	240.24	275.44	0.1759	5040.583	0.2111	11.1111	164	0.6884
9	220.89	478.48	478.48	6.6076	20.443	279.23	384.05	0.4646	3323.169	0.6033	5.6566	659	0.8658
10	166.46	448.1	410.13	6.3797	9.2025	364.29	609.62	0.3785	3936.985	0.1744	8.4828	137	0.8957
11	185.44	408.86	405.06	7.1392	5.8608	298.72	175.19	0.262	4775.983	0.0692	10.9900	59	0.6954
12	157.59	424.05	483.54	6.6835	3.1266	249.1	392.41	0.419	3447.658	0.0806	7.6768	61	0.6985
13	167.72	484.81	444.3	7.9747	15.5823	309.35	208.61	0.3532	4889.015	0.1866	11.1111	151	0.6998
14	223.42	418.99	481.01	6.0253	13.4557	254.42	242.03	0.3937	2943.098	0.4716	4.8485	495	0.7732
15	201.9	421.52	489.87	6.9114	14.9747	233.15	693.16	0.3633	3542.686	0.3482	6.6667	326	0.8163
16	194.3	481.01	440.51	7.1139	9.5063	358.97	367.34	0.4949	4153.039	0.1842	8.2828	177	0.8962
17	247.47	468.35	488.61	6.0759	4.3418	316.44	150.13	0.338	2850.170	0.1553	4.2424	179	0.7862
18	199.37	489.87	400	7.2911	7.0759	275.68	509.37	0.2013	4893.874	0.0773	9.6981	69	0.8489
19	187.97	460.76	454.43	6.8354	4.038	305.81	743.29	0.4696	3782.419	0.0915	7.4748	82	0.8971
20	182.91	483.54	431.65	7.8734	14.3671	318.21	718.23	0.3582	4972.935	0.1672	10.6533	140	0.8984
21	198.1	425.32	402.53	7.1646	21.0506	295.18	709.87	0.1253	4804.986	0.2324	9.6970	203	0.8673
22	158.86	467.09	467.09	7.4177	16.4937	257.96	534.43	0.4747	4137.657	0.3131	9.4950	234	0.7397
23	208.23	401.27	429.11	6.8101	17.1013	266.82	475.95	0.4797	4123.896	0.3296	7.8788	295	0.8483
24	195.57	422.78	474.68	6.962	24.6962	335.93	400.76	0.4443	3725.774	0.5617	7.2727	512	0.8868
25	212.03	420.25	422.78	7.9241	19.2278	362.52	551.14	0.4241	5231.738	0.2149	29.7487	204	0.9969
26	225.95	497.47	424.05	7.6456	7.9873	263.28	317.22	0.4848	4820.629	0.1102	8.8889	121	0.8289
27	152.53	455.7	449.37	6.2785	21.962	234.92	634.68	0.3177	3407.669	0.5311	7.6768	380	0.7067
28	181.65	463.29	426.58	6.2025	13.1519	236.69	342.28	0.4899	3556.548	0.3468	7.0707	304	0.7193
29	184.18	473.42	416.46	7.2405	15.2785	367.83	450.89	0.1658	4614.659	0.1851	9.6970	151	0.9104
30	206.96	465.82	463.29	7.8987	3.4304	353.66	542.78	0.2165	4555.234	0.0436	8.8889	40	0.9725
31	180.38	492.41	496.2	6.7089	8.5949	245.56	442.53	0.257	3252.619	0.2204	6.4647	178	0.7545
32	150	475.95	462.03	6.9873	23.7848	334.16	517.72	0.2975	3833.151	0.4468	8.8889	318	0.8029
33	238.61	454.43	417.72	6.5316	6.7722	337.71	684.81	0.2873	3920.651	0.1215	6.6667	129	1.0494
34	218.35	498.73	451.9	6.8861	13.7595	314.67	108.35	0.3076	3788.923	0.2678	6.8687	274	0.6656
35	222.15	427.85	425.32	7.5696	4.6456	261.5	668.1	0.1962	4826.271	0.0521	9.0909	51	0.8996
36	217.09	500	475.95	7.4937	16.1899	286.31	417.47	0.1506	4014.266	0.2593	7.4748	250	0.8780
37	163.92	403.8	446.84	6.2278	18.9241	341.25	325.57	0.2722	3450.967	0.4332	7.4748	330	0.7893
38	242.41	451.9	486.08	7.443	10.7215	371.38	191.9	0.2924	3914.552	0.1936	6.6667	210	0.8823
39	200.63	496.2	432.91	6.481	22.8734	256.19	375.7	0.2418	3679.468	0.4534	6.8687	413	0.7928

40	250	472.15	407.59	6.6329	18.0127	373.15	308.86	0.343	4119.411	0.3044	6.8687	353	1.0009
41	244.94	402.53	427.85	7.2658	15.8861	270.37	300.51	0.2215	4528.199	0.2052	7.8788	225	0.8644
42	196.84	491.14	436.71	6	1.9114	293.4	467.59	0.3127	3246.639	0.0511	5.8586	46	0.8578
43	151.27	441.77	477.22	6.5063	11.3291	272.14	141.77	0.1608	3344.963	0.2621	7.4748	186	0.5730
44	219.62	413.92	434.18	7.0633	8.8987	346.57	501.01	0.1051	4260.086	0.1248	7.8788	121	0.9830
45	203.16	493.67	497.47	6.3291	11.9367	366.06	425.82	0.2316	2957.817	0.3678	5.2525	342	0.9461
46	227.22	487.34	445.57	6.7342	11.6329	247.33	734.94	0.3278	3739.509	0.2359	6.4647	245	0.8940
47	232.28	445.57	435.44	7.7215	22.2658	243.78	617.97	0.3684	4784.199	0.2833	8.6869	303	0.8847
48	233.54	405.06	487.34	7.1899	7.6835	339.48	567.85	0.4342	3791.702	0.1671	6.6667	179	1.0201
49	205.7	434.18	469.62	6.4304	2.519	259.73	434.18	0.1304	3331.132	0.0586	6.0302	53	0.8237
50	186.71	415.19	448.1	6.1013	12.5443	307.58	751.65	0.2823	3304.914	0.3163	6.4647	266	0.8973
51	175.32	407.59	437.97	7.8228	6.4684	325.3	701.52	0.3835	4959.940	0.0774	11.1158	63	0.8864
52	215.82	417.72	439.24	6.1266	3.7342	343.02	292.15	0.3329	3389.984	0.0928	5.8598	91	0.8893
53	246.2	426.58	441.77	7.038	1.6076	277.45	333.92	0.4291	4104.419	0.0296	7.0707	34	0.8919
54	168.99	435.44	500	7.5949	10.1139	304.04	592.91	0.2671	3975.415	0.1734	8.8889	129	0.8302
55	214.56	482.28	430.38	6.3544	19.8354	312.9	760	0.1152	3604.122	0.3913	6.4647	382	0.9704
56	189.24	410.13	470.89	7.3165	18.3165	242.01	183.54	0.3481	4081.733	0.3152	8.3674	274	0.6567
57	190.51	406.33	460.76	8	12.2405	330.62	258.73	0.3228	4799.958	0.1489	10.0993	126	0.8032
58	156.33	488.61	472.15	6.0506	16.7975	302.26	225.32	0.3734	2989.252	0.5549	6.2626	438	0.6817
59	234.81	458.23	459.49	7.7975	18.6203	252.64	116.71	0.2468	4505.201	0.2459	8.1633	267	0.6563
60	161.39	469.62	453.16	7.0127	1	300.49	659.75	0.181	3952.535	0.0166	8.8889	12	0.8157
61	236.08	437.97	464.56	6.3038	17.4051	332.39	166.84	0.1456	3255.428	0.4269	5.2525	469	0.8052
62	239.87	416.46	498.73	7.2152	12.8481	282.77	350.63	0.1405	3680.391	0.2450	6.2626	262	0.8944
63	174.05	429.11	413.92	7.3418	14.0633	344.8	133.42	0.5	4818.338	0.2026	10.5051	184	0.6557
64	231.01	479.75	468.35	6.8608	17.7089	360.74	676.46	0.4139	3606.310	0.4173	6.2626	434	1.0623
65	248.73	464.56	420.25	6.7848	8.2911	268.59	250.38	0.1101	4098.008	0.1261	6.8687	139	0.8394
66	228.48	486.08	465.82	7.9494	23.481	321.76	358.99	0.4089	4519.534	0.3479	8.2828	345	0.9250
67	160.13	477.22	401.27	6.4051	14.6709	323.53	158.48	0.2772	4054.463	0.2430	9.0909	177	0.6474
68	204.43	474.68	408.86	6.557	20.1392	311.12	626.33	0.4595	4062.830	0.3904	7.6768	342	0.9278
69	241.14	439.24	458.23	7.3924	23.1772	327.07	584.56	0.1861	4198.322	0.3434	7.2727	372	1.0229
70	155.06	456.96	411.39	7.6709	2.2152	288.09	409.11	0.4392	5163.405	0.0258	12.3232	17	0.7419
71	224.68	494.94	482.28	7.7722	2.8228	281	643.04	0.3886	4167.537	0.0483	7.6768	48	0.9720
72	179.11	453.16	479.75	7.0886	21.6582	350.11	100	0.2266	3740.816	0.4113	7.8788	357	0.6071
73	191.77	444.3	491.14	6.4557	21.3544	291.63	526.08	0.1709	3161.939	0.5581	6.0606	508	0.8567
74	162.66	400	450.63	7.4684	9.8101	238.47	576.2	0.1354	4488.919	0.1244	10.3030	90	0.7282
75	209.49	450.63	493.67	7.6962	7.3797	289.86	283.8	0.4544	4061.957	0.1425	7.8788	142	0.8140
76	210.76	443.04	421.52	6.5823	25	284.54	125.06	0.3987	3951.531	0.4810	7.2727	480	0.6619
77	237.34	459.49	492.41	6.7595	5.557	319.99	726.58	0.2063	3327.546	0.1334	5.4546	140	1.0270
78	172.78	412.66	455.7	7.7468	19.5316	357.2	601.27	0.1557	4640.429	0.2331	10.3030	180	0.9033
79	177.85	432.91	494.94	6.6582	4.9494	369.61	559.49	0.1	3304.923	0.1157	6.6667	93	0.9225
80	193.04	446.84	456.96	7.8481	6.1646	273.91	267.09	0.1203	4634.633	0.0731	19.7980	62	0.7549
